# The association between colorectal cancer and prior antibiotic prescriptions: case control study

**DOI:** 10.1038/s41416-019-0701-5

**Published:** 2020-01-13

**Authors:** David Armstrong, Alex Dregan, Mark Ashworth, Patrick White, Chris McGee, Simon de Lusignan

**Affiliations:** 1 School of Population Health & Environmental Sciences, King’s College London, London, UK; 2 Department of Psychological Medicine, Institute of Psychiatry, Psychological, and Neurosciences, King’s College London, London, UK; 3 Department of Clinical and Experimental Medicine, Surrey University, Guildford, UK; 4 Royal College of General Practitioners (RCGP) Research and Surveillance Centre (RSC), London, UK

**Keywords:** Epidemiology, Public health

## Abstract

**Background:**

Antibiotic use over several decades is believed to be associated with colorectal adenomas. There is little evidence, however, for the effect of more recent antibiotic use on frequency of colorectal cancers.

**Methods:**

A case control study used the RCGP’s Research and Surveillance Centre cohort of patients drawn from NHS England. In all, 35,214 patients with a new diagnosis of colorectal cancer between 1 January 2008 and 31 December 2018 were identified in the database and were matched with 60,348 controls. Conditional logistic regression was used to examine the association between antibiotic prescriptions and colorectal cancer.

**Results:**

A dose-response association between colorectal cancers and prior antibiotic prescriptions was observed. The risk was related to the number and recency of prescriptions with a high number of antibiotic prescriptions over a long period carrying the highest risk. For example, patients prescribed antibiotics in up to 15 years preceding diagnosis were associated with a higher risk of colorectal cancer (odds ratio (OR) = 1.90, 95% confidence intervals (CI), 1.61–2.19, *p* < 0.001).

**Conclusions:**

Antibiotic use over previous years is associated with subsequent colorectal cancer. While the study design cannot determine causality, the findings suggest another reason for caution in prescribing antibiotics, especially in high volumes and over many years.

## Background

Accumulating evidence suggests that the human intestinal microbiota contributes to the aetiology of colorectal cancer (CRC), not only via the pro-carcinogenic activities of specific microorganisms but also via the influence of the wider microbial community.^1–7^ On the one hand, pro-inflammatory and DNA-damaging colonic bacteria and their metabolites are thought to contribute to colorectal carcinogenesis, while on the other hand, metabolites produced by other bacteria may exert a protective function against damage to the intestinal wall. This suggests that the balance of the organisms making up the gut microbiota may be a significant factor determining cell mutation and subsequent cancer risk.

Analyses of faecal samples from patients with CRC compared with controls have identified over and under representation of some bacterial species in the cancer patients, though results have not been consistent.^8–11^ The increasing list of potentially carcinogenic bacteria provides support for the hypothesis that the development of cancer is driven by mechanisms and/or pathways that are common to many bacterial groups rather than a single organism. These observations have led to suggestions that targeted antibiotics, probiotics or prebiotics may have a role to play in CRC prevention or treatment.^12,13^

Antibiotics are also known to produce dysbiosis in the microbiome and this disturbance may facilitate the sorts of cellular damage known to precede cancerous growths.^12,13^ Support for this hypothesis comes from the US Nurses’ Health Study, a cohort of over 16,000 women, where there was a clear association between prior antibiotic use and subsequent colorectal adenoma, a well-recognised precursor of carcinoma.^14^ Indeed, prior use of antibiotics, a common prescription in health care, provides a ‘natural experiment’ to explore the association between antibiotics and CRC risk. We therefore used a large clinical database with records of a colorectal cancer diagnosis together with preceding antibiotic use to test the hypothesis that the frequency of antibiotic prescribing over several years increases the risk of subsequent colorectal cancer diagnosis.

## Methods

### Data

A retrospective case-control study was implemented within a cohort of patients registered in the UK’s Royal College of General Practitioners’ (RCGP) Research and Surveillance Centre (RSC). The RCGP RSC extracted pseudonymised data for a cohort of around 2 million patients registered in over 260 participating general practitioner (GP) practices at the time of this study, and represents the principal primary care public health surveillance data used by Public Health for England (PHE). Almost everyone in the UK is registered with a general practice, which provides and coordinates most of their health care. General practices have been computerised since the 1990s recording key clinical data using Read codes. Treatment records are generally complete as electronic prescribing is widely used. Patients in the database have been found to be representative of the UK population in terms of gender and age, with slight under-representation of people from a minority ethnic background and less deprived communities.^15^ RCGP RSC practices have had long-term feedback about data quality, most recently via a dashboard.^16^

### Study population

The study population consisted of all patients with newly diagnosed CRC between 1 January 2008 and 31 December 2018 in the RSC database. Data were extracted from the RSC in April 2019. The cohort of CRC patients was identified using Read codes selected in our previous research.^17^ Cases were all patients, aged 18 and over, with a first ever record of CRC (the index date) during the study period. All cases had at least 12 months of clinical record before the diagnosis of CRC. Cases were matched on year of birth (within 2 years to increase the probability of identifying four controls for each case), gender, index date and the same general practice with up to four controls. Controls were assigned the index date of diagnosis of the matched case and were patients without a CRC diagnosis who were alive, registered with the practice and with at least 12 months of clinical record prior to the index date. Patients with a diagnosis of inflammatory bowel disease were excluded from the study.

### Exposures

The number of antibiotic prescriptions issued to patients represented the primary variable of interest. RCGP RSC has reliable antibiotic prescribing since 2003 so the maximum exposure was up to 15 years. Antibiotic drugs included were those in the British National Formulary (BNF) chapter 5.1 excluding anti-tuberculous and anti-leprotic drugs. All antibiotic prescriptions after the study start date (defined as the earliest of the registration date, or record of antibiotic prescribing) and before the index date for CRC were included. Three different exposure variables were developed, namely cumulative number of prescriptions, specificity (subtypes) of antibiotic drugs and date of last prescription. For the cumulative number of prescriptions, patients were grouped according to the number of relevant antibiotic prescriptions (0, 1, 2, 3–4, 5–10, >10) issued over specified total time periods before the index date (within 1 year, 5 years and ‘ever’, that is up to the maximum recorded years). Two classes of antibiotic were chosen for specific analysis across the three time periods, penicillins (BNF 5.1.1) on the grounds they were most frequently prescribed^18^ and quinolones (BNF 5.1.12) given their broad spectrum of activity. The last date of antibiotic prescribing was calculated as the time gap from the last date of a relevant drug prescription in relation to the index date for CRC (e.g. 1 year, 2 years, 3–5 years, 5–10 years and 10 or more years).

### Covariates

Factors known or proposed to impact the association between antibiotic prescribing with CRC were a priori included as covariates in the analyses. These included deprivation (quartiles of the Index of Multiple Deprivation 2010), ethnicity (White, Black, Asian, Chinese or Other), smoking (never, ex-smoker and current smoker), body mass index (BMI); <18.5, 18.5–24.9, 25–29.9, >29.9 kg/m^2^, total cholesterol (0–4, 4–<5, 5–<6, 6–15 mmol/l) and a series of long-term conditions as markers of frailty included as binary variables: coronary heart disease (CHD), stroke, chronic obstructive pulmonary disease (COPD), liver disease, type 2 diabetes, depression and dementia. For each covariate the value closer to the study baseline (start date) was considered.

### Statistical analysis plan

Descriptive analyses were used to compare baseline differences between cases and controls. Participants were followed-up from the study start date, that is from the beginning of possible antibiotic recording, to the study end date, the index date when CRC was diagnosed. Conditional logistic regression was employed to estimate odds ratios and 95% confidence intervals (CIs) for the association between CRC and previous antibiotic prescriptions. The main analysis modelled antibiotic prescription with overall CRC risk by including all drug classes (overall antibiotics) within the same estimation model across three time periods, 1 year, 5 years, and ‘ever’ prior to the index date. Separate models were estimated for type of antibiotic drugs (penicillins and quinolones) and last date of antibiotic prescribing. The analyses adjusted for the main hypothesised confounding variables described above. The analyses excluded all antibiotic prescriptions issued within 6 months before diagnosis to reduce the risk of protopathic bias. A sensitivity analysis was also carried out by excluding prescriptions issued 12 months before the diagnosis to examine a longer protopathic period. Data for study covariates were not available for all participants and we implemented multiple imputation with chained equation (10 imputed datasets) to address missing data concerns. Following Rothman^19^ and Greenland,^20^ the analyses did not adjust for multiple comparisons. The analyses were implemented in Stata version 15. (StataCorp, College Station, TX).

## Results

**Fig. 1:**
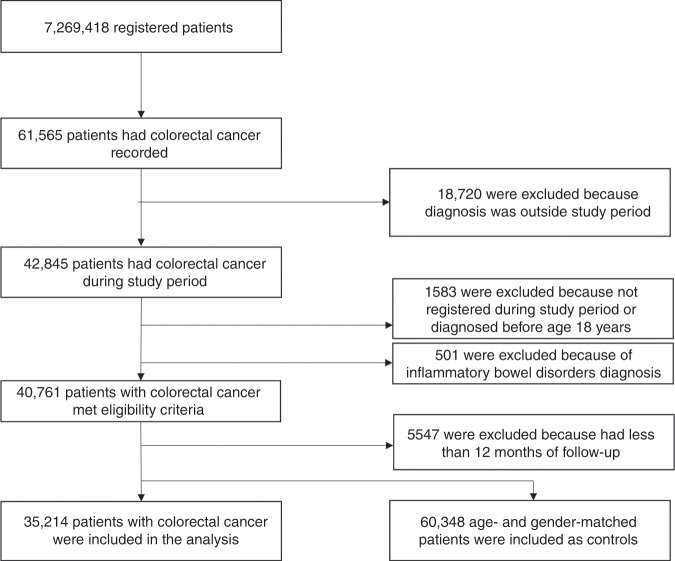
Flowchart describing the study sample selection.

Between 1 January 2008 and 31 December 2018, a total of 35,214 patients with a new diagnosis of colorectal cancer (median follow-up(*m*) = 6 years, interquartile range (IQR) = 4–7) were identified in the database that were matched with 60,348 controls (*m* = 6 years, IQR = 3–9 years) (Fig. 1). Table 1 shows the distribution of matching variables and co-variates between colorectal cancer cases and matched controls. The study population had a mean age of 69 years at colorectal cancer diagnosis.

**Table 1:** Distribution of baseline characteristics between colorectal cancer cases and controls matched by age, gender, index date and general practice.

	Colorectal cancer (*N* = 35,214)	Controls (*N* = 60,348)
Female	17,720 (50)	30,282 (50)
Age—Mean(sd)	68.68 (12.81)	67.38 (14.24)
White ethnicity	26,791 (95)	42,010 (92)
Deprivation (Median, IQR)	13 (8,23)	14 (8.25)
BMI, Mean(sd)	27.41 (5.31)	27.13 (5.41)
Smoking	6857 (22)	11,327 (25)
Total cholesterol Mean(sd)	5.08 (1.15)	5.06 (1.48)
Coronary heart disease	4538 (13)	7961 (13)
Stroke	1345 (4)	2741 (5)
COPD	1618 (5)	3036 (5)
Liver disease	315 (1)	482 (1)
Diabetes	4030 (11)	6085 (10)
Depression	7124 (20)	10,810 (18)
Dementia	79 (0)	666 (1)
Colonoscopy	5627 (16)	2958 (5)

Figures are frequencies and percentages, unless otherwise specified.

*sd* standard deviation, *IQR* interquartile range, *BMI* body mass index, *COPD* chronic obstructive pulmonary disease

**Fig. 2:**
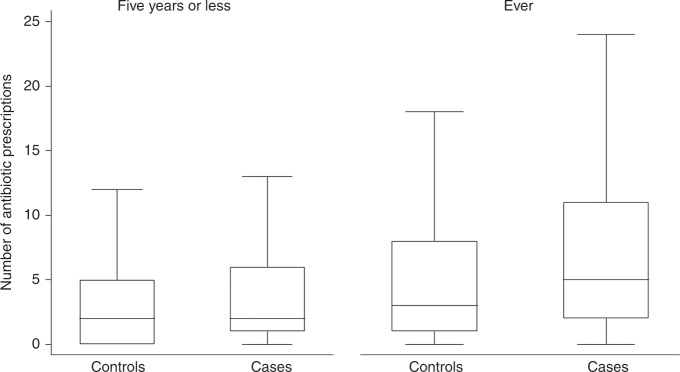
Boxplot showing the number of antibiotic prescriptions, stratified by timing in relation to the index date.

Patients with colorectal cancer showed higher median number of antibiotic prescriptions compared with their matched controls across the 5-years and ever time-intervals (Fig. 2). For instance, the median number of antibiotic prescriptions for cases was 5 (IQR 2–8), and for controls was 3 (IQR 1–8).

**Fig. 3:**
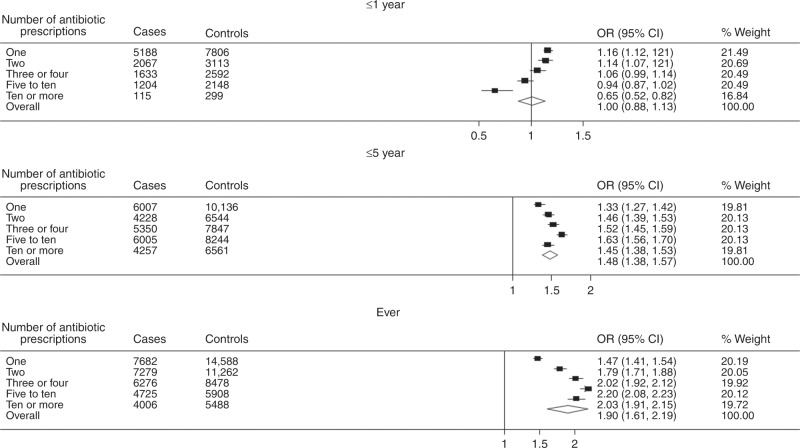
Conditional logistic regression results for antibiotic prescriptions association with colorectal cancer risk.

Figure 3 shows an adjusted 90% (odds ratio (OR) = 1.90, 95% confidence intervals (CI), 1.61–2.19, *p* < 0.001) overall increased probability of antibiotic prescribing over the whole study period, following subjects up to 15 years. There was also a gradual increase in colorectal cancer risk with the number of antibiotic prescriptions. For instance, the probability of colorectal cancer increased from 47% (OR = 1.47, 95% CI, 1.41–1.54, *p* < 0.001) for one antibiotic prescription to 103% (OR = 2.03, 95% CI, 1.91–2.15, *p* < 0.001) after more than 10 prescriptions. A similar pattern, though lower estimates, was observed within the 5-year time interval. The 1-year time interval-based findings showed an association with colorectal cancer, the risk, however, declined with the number of prescriptions.

**Fig. 4:**
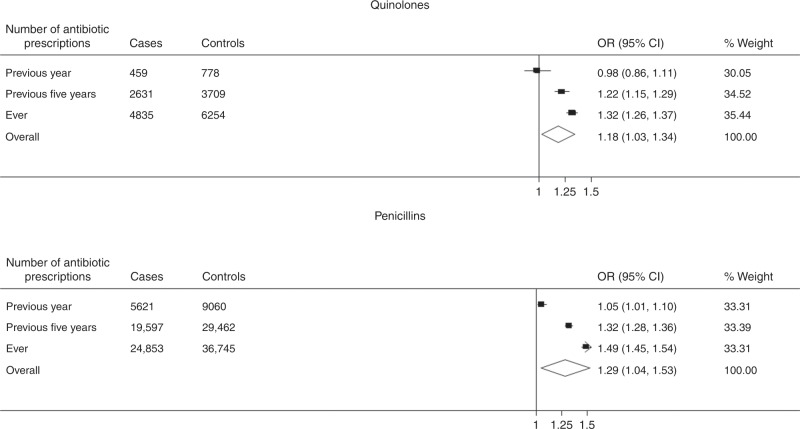
Conditional logistic regression results for antibiotic types association with colorectal cancer risk.

The analyses stratified by two major antibiotic groups (Fig. 4) indicated that both quinolone or penicillin prescribing were associated with an increased probability of colorectal cancer (OR = 1.18, 95% CI, 1.03–1.34, *p* < 0.001 and OR = 1.29, 95% CI, 1.04–1.53, *p* < 0.001, respectively). The strength of the association between both quinolones and penicillin with colorectal cancer increased with an extended exposure interval.

Analyses that explored the association between the time since the last antibiotic prescribed with the risk of colorectal cancer revealed a positive association between the last antibiotic prescription and a CRC diagnosis whatever the time interval since the last prescription (Table 2). The patterns for both quinolones and penicillin were similar.

**Table 2:** Results of conditional logistic regression showing the association between the last prescription prior to study index date and colorectal cancer diagnosis.

	Antibiotics	Quinolones	Penicillin
Last prescription			
Previous year	1.17 (1.13–1.21)	1.11 (1.01–1.22)	1.06 (1.02–1.09)
≥2 years	1.05 (1.01–1.10)	1.01 (0.89–1.13)	1.07 (1.03–1.12)
≥3 years	1.07 (1.01–1.13)	1.12 (0.99–1.27)	1.12 (1.06–1.18)
≥5 years	1.09 (1.04–1.15)	1.22 (1.13–1.32)	1.23 (1.17–1.29)
≥10 years	1.16 (1.03–1.31)	1.32 (1.16–1.50)	1.19 (1.07–1.32)

Note: Analyses adjusted for BMI, smoking, ethnicity, deprivation, cholesterol, CVD, type II diabetes, COPD, depression, dementia, and liver disorders

Sensitivity analyses that extended the protopathic bias interval to 12 months prior to the index date, revealed similar patterns to the main study findings (Supplementary material, Fig. S1) confirming the observed dose-response association. Similar findings were observed with respect to quinolone (1.34, 95% CI, 1.28–1.40) and penicillin (1.52, 95% CI, 1.49–1.57) prescribing. These analyses were restricted to antibiotic prescribing over the full study period.

## Discussion

The study reported here found a strong association between colorectal cancers and prior antibiotic prescriptions. The strength of the association increased with both the duration of the exposure and, broadly, with the number of prescriptions. Similar patterns emerged with regards to quinolones and penicillins prescribing. A significant association was observed even when quinolones and penicillins prescribed stopped 5 years prior to CRC, implying an enduring adverse impact of antibiotic prescribing similar to the effects observed for ex-smokers and lung cancer (i.e. ex-smokers are several times a higher risk of lung cancer even 10–15 years after quitting smoking compared with never smokers).

Results from the US Nurses’ Health Study found an increase in colorectal adenomas with antibiotics prescribed over the preceding four decades.^14^ The relationship was dose-related with longer periods of antibiotic consumption leading to higher rates of adenomas. If antibiotic prescriptions in earlier decades predisposes to adenomas might the more recent use of antibiotics lead those lesions to become malignant? Whereas the Nurses’ Health Study found no relationship between antibiotic use in the 4 years immediately prior to diagnosis and the occurrence of adenomas, this current study, examining colorectal cancers as the endpoint, did show an association between antibiotic use and cancers. The highest odds ratios were observed in periods furthest from the diagnosis and after use of high numbers of antibiotic prescriptions suggesting an accumulation model for cancer emergence.

A number of other studies examining shorter periods of antibiotic exposure support an association between prior antibiotic use and colorectal cancer, albeit with smaller effect sizes. Kilkkinen et al.^21^ identified all cancers diagnosed between 1998 and 2004 in a Finnish clinical cohort and linked these to antibiotics prescribed in an earlier 3-year period between 1995 and 1997. Several cancers showed a significant association with the previous number of antibiotic prescriptions (the relative risk for colon cancer was 1.15, CIs, 1.04–1.26). Bousi et al.,^22^ in a case control study using the THIN (The Health Improvement Network) database derived from primary care records in the UK, found that penicillin use was associated with increased diagnosis of colorectal cancer with the risk being greatest in the immediate year prior to diagnosis—a finding they attributed to possible protopathic bias (i.e. ‘reverse causality’) because of prescribing for early, non-specific disease manifestations. Antibiotic use before a year prior to diagnosis, however, was not reported as having a significant association with subsequent cancer. Dik et al.^23^ also used a case control design to study the effect of antibiotic use between 1 and 6 years prior to colorectal cancer diagnosis in a Dutch health insurance database. They found an adjusted odds ratio of 1.26 (CIs 1.11–1.44) for a high (≥8) number of antibiotic prescriptions in the designated 5-year period prior to diagnosis.

The study reported here used a wider range of years of antibiotic exposure, examining 1 year prior, 5 years prior or ‘ever’ (up to about 15 years prior). Although there are reports that the microbiome recovers rapidly from a short challenge with antibiotics this seems dependent on the type of antibiotic.^24,25^ In this study, moreover, those patients with repeated antibiotic prescriptions over many years seemed most at risk of CRC. High numbers of antibiotic prescriptions, however, in the year immediately preceding diagnosis (allowing for a 6-month protopathic period) appeared to have a possible protective effect against CRC. If one hypothesis for the long-term effect of antibiotics is microbiome dysbiosis, it is possible that in the short-term, intensive high-volume antibiotics—more than 10 antibiotic prescriptions in what amounts to a 6-month period is likely similar to a continuous course—may remove specific organisms that have been suggested to have a carcinogenic effect. This might indicate that specifically targeted narrow spectrum antibiotics may have a different effect compared with broad spectrum ones.

Several studies have suggested that the ‘symptomatic’ pre-diagnosis period in colorectal cancer is longer than had been assumed with various non-specific symptoms, such as fatigue and malaise, occurring in the months prior to diagnosis.^26,27^ These symptoms may lead to both increased GP consultations (Renzi et al.^28^ observed an increase in the consultation rate six months prior to diagnosis) and a greater likelihood of receiving a prescription for minor infections. We addressed this potential protopathic bias in all our analyses by excluding antibiotic prescriptions in the immediate 6 months prior to the CRC diagnosis. To allow for the possibility that the protopathic period was longer than 6 months we conducted a sensitivity analysis that excluded all prescriptions in the year preceding diagnosis: this showed no reduction in the long-term association of antibiotics with colorectal cancer.

It is important to stress that a case control study can only show associations, not causality, and may be subject to residual confounding. The study adjusted for confounders documented by previous studies to influence the association between antibiotic prescribing and CRC incidence but cannot rule out unknown confounders. Family history of CRC represents an important unmeasured confounder in our study, as this information is poorly captured in clinical records. However, 99% of our study cases were over 40-years-old at the time of CRC diagnosis, which limited the impact that biological predisposition may have on CRC prevalence. Antibiotics may have been given for gastro-intestinal complaints (we excluded patients recorded as having inflammatory bowel disease given its known association with CRC^29^), but most antibiotics are prescribed for upper respiratory tract infections.^18^ The RCGP RSC database only included prescriptions issued in general practice though most antibiotic prescribing is known to be from primary care.^30^ Our exposure variable was the number of antibiotic prescriptions, but we had no information on the dose or duration of each course and whether this differed between the cases and controls. We have no direct evidence that prescribed antibiotics were actually consumed by patients, however, non-adherence is likely to be common to both cases and controls and unlikely to bias the observed estimates.

In conclusion, earlier studies have indicated an association between antibiotic use and colorectal cancers and adenomas. Using a case control design, our study adds weight to these findings and explores the importance of the ‘susceptible’ time period before the cancer diagnosis as well as the effect of the number of antibiotic prescriptions. The strongest associations were found between CRC diagnosis and prescribed antibiotics over a long time period and also with the overall frequency of antibiotics prescribed—though high numbers of prescriptions over a shorter time period may have provided protection though a different mechanism. Reassuringly the ‘risks’ from taking an antibiotic seems to decline over time; if the oncogenic mechanism is damage to the gut microbiome this might indicate it is given the opportunity for recovery. Nevertheless, the study adds to the emerging hypothesis that antibiotic consumption is related to the occurrence of colorectal cancer and suggests a further reason for caution in antibiotic prescribing.

## Supplementary information

Figure S1

## Data Availability

Permission to access the data used in this study can be obtained from the RCGP’s Research and Surveillance Centre (https://www.rcgp.org.uk/clinical-and-research/our-programmes/research-and-surveillance-centre.aspx).
